# The Outcome of Metabolic and Bariatric Surgery in Morbidly Obese Patients with Different Genetic Variants Associated with Obesity: A Systematic Review

**DOI:** 10.3390/nu16152510

**Published:** 2024-08-01

**Authors:** Marija Zafirovska, Aleksandar Zafirovski, Tadeja Režen, Tadeja Pintar

**Affiliations:** 1 Faculty of Medicine, University of Ljubljana, Vrazov trg 2, 1000 Ljubljana, Slovenia; mz3061@student.uni-lj.si (M.Z.); aleksandar.zafirovski@sb-je.si (A.Z.); 2 Association of General Practice/Family Medicine of South-East Europe (AGP/FM SEE), St. Vladimir Komarov No. 40/6, 1000 Skopje, North Macedonia; 3 General Hospital Jesenice, Cesta maršala Tita 112, 4270 Jesenice, Slovenia; 4 Clinical Institute of Radiology, University Medical Centre Ljubljana, Zaloška cesta 7, 1000 Ljubljana, Slovenia; 5 Centre for Functional Genomics and Bio-Chips, Institute of Biochemistry and Molecular Genetics, Faculty of Medicine, University of Ljubljana, Zaloška cesta 4, 1000 Ljubljana, Slovenia; 6 Department of Abdominal Surgery, University Medical Centre Ljubljana, Zaloška cesta 2, 1000 Ljubljana, Slovenia

**Keywords:** obesity, morbid obesity, metabolic and bariatric surgery, genetic variant, polymorphism, single nucleotide polymorphism

## Abstract

Metabolic and bariatric surgery (MBS) effectively treats obesity and related comorbidities, though individual responses vary. This systematic review examines how genetic variants influence MBS outcomes in morbidly obese patients. A comprehensive search in PubMed, Embase, Medline, and the Cochrane Library identified 1572 studies, with 52 meeting the inclusion criteria. Two reviewers independently filtered and selected studies, including relevant cross-references. Research focused on polymorphisms in genes such as UCP2, UCP3, 5-HT2C, MC4R, FKBP5, FTO, CAT haplotypes, LYPAL-1, PTEN, FABP-2, CNR1, LEP656, LEP223, GLP-1R, APOA-1, APOE, ADIPOQ, IL-6, PGC1a, TM6SF2, MBOAT7, PNPLA3, TCF7L2, ESR1, GHSR, GHRL, CD40L, DIO2, ACSL5, CG, TAS2R38, CD36, OBPIIa, NPY, BDNF, CLOCK, and CAMKK2. Most studies explored associations with post-surgery weight loss, while some examined metabolic, cardiovascular, taste, and eating behavior effects as well. Understanding the role of genetic factors in weight loss and metabolic outcomes post-MBS can help tailor personalized treatment plans for improved efficacy and long-term success. Further research with larger sample sizes and extended follow-up is needed to clarify the effects of many genetic variants on MBS outcomes in morbidly obese patients.

## 1. Introduction

Obesity is a significant risk factor for several of the leading causes of mortality worldwide, including cardiovascular disease, stroke, diabetes, and various forms of cancer. According to the latest data of the Institute of Health Metrics and Evaluation (IHME), which are from 2021, obesity is the sixth leading risk factor for death in the world. In 2021, obesity was accountable for 129 million (95% UI 56.0–202) disability-adjusted life years (DALYs) and 3.71 million (1.85–5.66) deaths worldwide [1]. Studies have proven that obesity leads to the development of obesity-related diseases, such as the following: diabetes mellitus type 2 (T2D), metabolic syndrome, hypertension, hyperlipidemia, chronic kidney disease, cardiovascular disease, obstructive sleep apnea, osteoarthritis, malignancies, and metabolic dysfunction-associated steatotic liver disease (MASLD) [2,3,4,5]. Metabolic syndrome, a cluster of metabolic disorders including central obesity, high blood pressure, elevated fasting glucose levels, atherogenic dyslipidemia, and insulin resistance, further exacerbates the health risks associated with obesity. Individuals with metabolic syndrome are at heightened risk for cardiovascular disease, stroke, and T2D, underscoring the urgent need for effective preventive measures and management strategies [6].

Weight loss interventions such as diet, exercise, and metabolic and bariatric surgery (MBS) reduce all-cause mortality in obese adults [7]. MBS is linked to a decreased risk of premature death and the development of obesity-related diseases at the population level, and is considered the most effective treatment option for obesity [8,9]. Older MBS procedures like vertical banded gastroplasty (VBG) have been replaced by safer and more effective operations, with sleeve gastrectomy (SG) and Roux-en-Y gastric bypass (RYGB) now accounting for approximately 90% of all procedures performed worldwide. Other less common procedures include adjustable gastric banding (AGB or LAGB), biliopancreatic diversion (BPD) with duodenal switch, and one-anastomosis gastric bypass (OAGB), with most MBS now performed using minimally invasive techniques [10,11]. According to the newest guidelines by the American Society of Metabolic and Bariatric Surgery (ASMBS) and the International Federation for the Surgery of Obesity and Metabolic Disorders (IFSO), MBS is strongly recommended for patients with a BMI ≥35 kg/m^2^ regardless of the presence, absence, or severity of comorbidities; it is recommended in patients with T2D and a BMI ≥30 kg/m^2^ and should be considered in patients with a BMI 30–34.9 kg/m² who have not achieved significant or sustained weight loss or improvement in comorbidities through non-surgical methods. The new guidelines also recommended modifying the BMI thresholds for the Asian population, since traditional BMI thresholds do not apply equally to all populations. In the Asian population, BMI >25 kg/m² indicates clinical obesity and MBS should be considered for individuals with a BMI >27.5 kg/m² [11]. An Expert Modified Delphi Consensus conducted in 2023 concluded that intra-gastric ballooning (IGB) is suitable for Class I obesity regardless of the presence, absence, or severity of comorbidities. SG and RYGB were recommended as viable treatment options for patients with Class I and II obesity, as well as patients with T2D and BMI of ≥30 kg/m². OAGB was deemed suitable for patients with Class II obesity and T2DM regardless of the presence, absence or severity of comorbidities [12]. MBS induces weight loss and remission of obesity-related diseases through several biological mechanisms, many of which are still not completely understood [13,14]. Although MBS is highly effective for long-term weight loss in morbid obesity, about 20% of patients fail to maintain over 50% excess weight loss. Twin and close-relative studies suggested a genetic component to weight loss, indicating that genetic factors may influence the outcomes of MBS [15].

Modern technology and genome-wide association studies (GWASs) have opened a whole new world of theories that allow us to tap into the genetic background of obesity and obesity-related diseases. The genetics of obesity has two distinct forms—syndromic and non-syndromic. The non-syndromic form consists of monogenic obesity (POMC, NPY, LEP, LEPR, MC3R, MC4R, FTO, PC1, GHSR, etc.) and polygenic obesity (UCP1, UCP2, UCP3, ADRB1, ADRB2, ADRB3, SLC6A14, etc.) [16]. Researches in the past decade have been focusing on genes as well as single nucleotide polymorphisms (SNPs) and their association with weight loss, eating behavior, biochemical markers, and cardiovascular and metabolic parameters after MBS [17]. The aim of this systematic review is to examine how genetic variants influence MBS outcomes in morbidly obese patients.

## 2. Materials and Methods

The Preferred Reporting Items for Systematic Reviews and Meta-Analyses (PRISMA) guidelines were used for this study. A systematic review protocol was constructed to describe the rationale and hypothesis and to define the methodology, and it was registered under the registration number INPLASY202470020.

### 2.1. Selection Criteria

The inclusion criteria for performing the search and selection were the following: studies conducted on adult patients aged 18 years or older diagnosed with morbid obesity and undergoing MBS, studies with a defined genetic variant/polymorphism of interest, and availability of follow-up data after MBS. We excluded studies on non-human subjects (animal studies), observational studies without a pre-surgery and post-surgery comparison (cross-sectional studies), reviews, letters to the editor and preprints, studies that are not available in the English language, and studies that are not freely available.

### 2.2. Search Strategy

An extensive literature search was performed in PubMed, Embase, Medline, and Cochrane library to identify eligible studies. The search was repeated 4 times and the date of the last search is 19.06.2024. Additional searching of gray literature or hand searching was not performed.

The used search terms were the following: (exp obesity/OR exp morbid obesity/OR exp diabetic obesity/) AND (exp bariatric surgery/) AND ((genetic variant.mp. OR exp genetic variability/) OR (exp gene/) OR (exp single nucleotide polymorphism/ OR exp genetic polymorphism/ OR polymorphism.mp)).

All the search results were summed up and duplicates were removed. For the first selection, two reviewers screened potentially relevant articles based on the title and abstract and according to predefined eligibility criteria. For the second selection, full articles were read to determine further inclusion. Cross-references of relevant studies and reviews were screened.

### 2.3. Data Extraction

Data from the included studies were extracted independently by two reviewers using a standardized data extraction form. The form included data on the study characteristics (author, year, country), sample size, patient demographics, details of the bariatric surgery performed, genetic variants or polymorphisms examined, follow-up duration, main outcomes measured (e.g., weight loss, metabolic parameters), and the association between the outcomes and the genetic variant/polymorphism. Discrepancies between reviewers were resolved through discussion or consultation with a third reviewer.

### 2.4. Data Synthesis

For this type of etiology/risk review, a narrative synthesis approach was used to analyze the data. The findings were summarized by integrating the results across studies by design, population, demographic data and outcomes without statistical pooling. Key findings were grouped according to the genetic variant/polymorphism and the type of bariatric surgery. Patterns and differences in outcomes were highlighted, and the potential influence of factors such as patient demographics and follow-up duration were discussed. The quality and risk of bias of the included studies were also assessed and considered in the interpretation of the results.

## 3. Results

**Figure 1:**
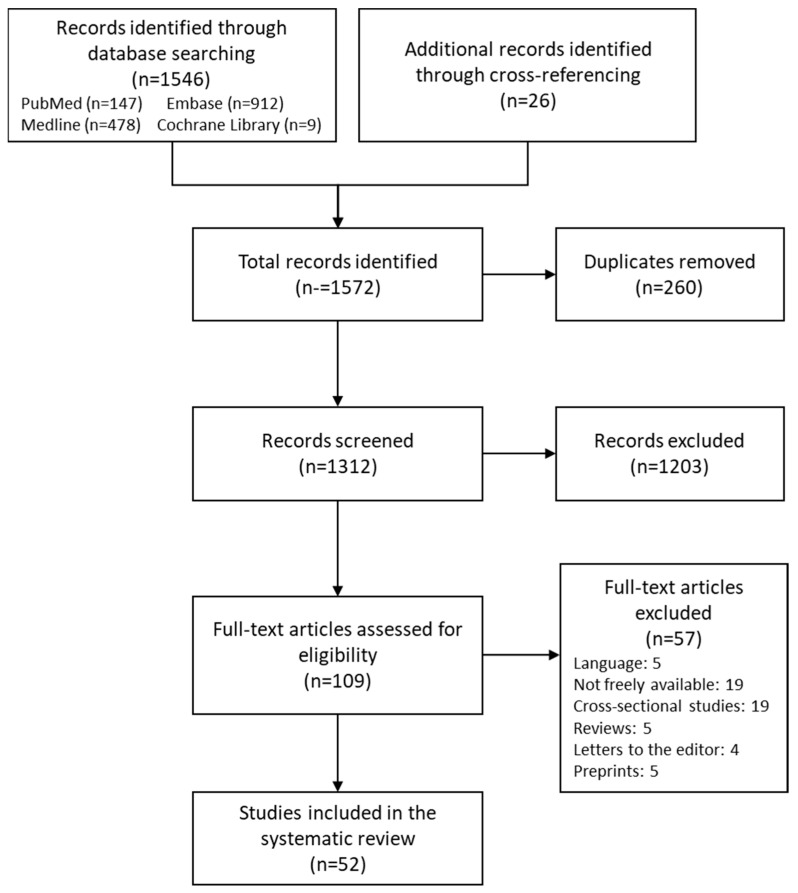
Flow diagram.

A total of 1572 studies were identified from which 260 were duplicates. After the first selection, 109 studies were selected, and after the second selection, only 52 were determined eligible to be included in qualitative synthesis (See Figure 1). Results were organized in a table according to the gene and polymorphism in order to compare them more efficiently (See Table 1).

### 3.1. Uncoupling Protein 2 Gene (UCP2) and Uncoupling Protein 3 Gene (UCP3)

Eight studies about UCP2 were found. For the rs659366 polymorphism, two studies with 164 patients that underwent LAGB and 150 that underwent RYGB showed that the A allele was associated with increased weight loss after 6 and 12 months, respectively [18,19]. Another study performed on 351 patients that underwent RYGB showed no association with % of excess weight loss (%EWL) after 12 months [20]. On the other hand, a study performed on 186 patients that underwent RYGB showed that the A/A genotype is associated with higher BMI, more excess weight, and lower %EWL 18 months after MBS. Patients with the Ins allele had a smaller decrease in BMI 12 months after MBS compared to those with the Del/Del genotype [21].

Regarding the rs660339 polymorphism, three studies concluded that the T allele was associated with weight loss after 6–24 months if the bariatric procedure was LAGB or RYGB, while there were no associations with BMI changes after laparoscopic mini-gastric bypass (LMGB) [19,22,23]. On the other hand, other studies performed on Brazilian patients showed no association with weight loss 12 months and 3 years after RYGB [20,24]. Nicoletti et al. concluded that genetic variants in the UCP2 gene were associated with dietary consumption after RYGB. It was shown that 1 year after MBS patients with rare variants in rs660339 and rs659366 had higher energy and carbohydrate intake. Patient with rare variants for Ala55Val had a higher protein consumption, as well [25].

Two studies found no association between UCP3 rs1800849 polymorphism and weight loss after MBS (RYGB and BPD) [20,26].

### 3.2. 5-Hydroxytryptamine Receptor 2C Gene (5-HT2C)

5HT2C gene rs3813929 polymorphism (more specifically, the TT genotype) predicted greater body weight loss after RYGB surgery. Post-surgery responsiveness may be related to the control of satiety, involving the interaction between a hormone (serotonin) and its receptor (5-HT2C) [20].

### 3.3. Melanocortin 4 Receptor Gene (MC4R)

Eight studies about MC4R were found. Women carriers of rs17782313 presented a higher pre-surgical BMI and did not attain a minimum BMI as low as women in the TT group. Furthermore, carriers of the rs17782313 polymorphism exhibited a significantly lower likelihood of achieving a non-obesity BMI compared to non-carriers [27]. On the other hand, in a study on 169 patients undergoing RYGB (78 women and 23 men), no significant association was found between the MC4R polymorphism and post-surgical weight and BMI [28].

Two studies showed that heterozygous MC4R mutations (NM_005912.2 c. 812G>T (p. Cys271Phe, NM_005912.2 c. 919C>T (p. Gln307stop), NM_005912.2 c. 706C>T (p. Arg236Cys), functional mutations, rs2229616, rs52820871, rs17782313, rs34114122) did not affect weight loss and body fat mass (FM) after 12 months of RYGB [29,30]. Another study confirmed that MC4R defects (rs52820871, NM_005912.2 c.594C>T (p. Ile198Ile)) are not associated with a higher complication rate following LAGB [31].

Regarding rs52820871, a study determined that I251L carriers have lower levels of baseline glucose and insulin levels leading to reduced risk of T2D. They are predisposed to better clinical outcome and better weight loss during diet and RYGB [32].

Carriers of MC4R gene variants, in contrast to matched severely obese patients without gene abnormalities and binge eating, exhibited an aggressive form of binge eating disorder associated with the metabolic syndrome and gastrointestinal pathology, with poorer response to massive sustained weight loss from food restriction after LAGB [33]. A study on 300 patients showed that rs79783591 mutation increases obesity risk, but one functional MC4R copy allows short-term weight loss from dietary restriction, phentermine, and RYGB [34].

### 3.4. FK506-Binding Protein 5 Gene (FKBP5)

Two studies on FKBP5 rs1360780 showed that the T allele is associated with weight loss. A study on 42 Brazilian obese patients showed that carriers of the T allele showed significantly lower weight loss compared to C/C genotype patients 12 to 14 months after MBS. T-allele carriers also experienced an earlier plateau in weight loss post-surgery [35]. Another study in 151 obese patients showed an association between the FKBP5 rs1360780 variant and sex, age, and type of surgery. Male T-allele carriers had higher BMI 24 months after MBS compared to non-carriers, particularly among those who underwent SG. Age also impacted weight loss, with older T-allele carriers exhibiting less favorable outcomes [36].

### 3.5. Fat Mass and Obesity-Associated Protein Gene (FTO)

Eight studies about FTO were found. One study indicated that the minor allele (G) of the FTO rs16945088 was associated with 3 kg less weight loss compared with common allele homozygotes (A/A), particularly in banding surgery patients, but not in gastric bypass subjects [37].

Studies examining the rs9939609 variant of the FTO gene in the context of MBS provided mixed results regarding its influence on weight loss. Weight loss progressed differently in obese carriers of the FTO gene variant rs9939609 after RYGB. The rs9939609 variant (AA or AT genotypes) did not affect weight loss until two years post-surgery, after which weight loss diminished and weight regain increased [39]. Conversely, other research concluded that FTO genotypes did not significantly influence weight loss two years post-RYGB [42]. This might be due to the pre-surgery vitamin D levels, which were proven to have influence on subsequent weight loss, especially in carriers with two copies of the A allele [41]. Additionally, one study reported higher weight loss at three months in TT carriers of rs9939609, with no significant differences at nine and twelve months post-BPD, but with a significant improvement in biochemical parameters and cardiovascular comorbidities [38]. Another study showed that there was no association between the FTO gene and weight loss 6 months after LSG [40]. However, in patients undergoing RSG, those without the A allele experienced better anthropometric and insulin level improvements, reducing diabetes risk after 12 months [43]. These findings suggest that the impact of the FTO rs9939609 variant on weight loss post-MBS varies and may depend on surgery type and other factors like vitamin D levels.

The rs9939609 and rs1421085 variants were linked to higher post-surgery weight, while rs9930506 and rs1421085 were associated with lower total body weight loss (TBWL) after MBS. Additionally, patients carrying these three polymorphisms exhibited elevated insulin levels and higher HOMA-IR, indicating increased insulin resistance [28].

### 3.6. Catalase (CAT) Haplotype

Variants in the antioxidant enzyme catalase are linked to hypertension, dyslipidemia, and diabetes. The CAT1 haplotype, comprising homozygous carriers of CATH1 [−844 G, −89 A, −20 T], contrasts with the CAT2 haplotype, which includes heterozygous carriers (CATH1/CATH2) and homozygous CATH2 [−844 A, −89 T, −20 C]. CAT haplotypes may contribute to the variability in metabolic and cardiovascular improvements observed during post-MBS follow-up. Specifically, the CAT1 haplotype appeared to display a more favorable effect on metabolic and cardiovascular parameters following MBS [44].

### 3.7. Lysophospholipase-like 1 Gene (LYPLAL1)

A study of 251 obese patients who underwent RYGB surgery found that those with the homozygous T allele of SNP rs4846567 near the LYPLAL1 gene had a 7% higher excess BMI loss (EBMIL) compared to other genotypes. These TT-allele carriers also exhibited better eating behavior outcomes, including lower Hunger and Disinhibition scores and higher Cognitive Restraint scores. Additionally, patients with the lowest Hunger scores had a 32% greater EBMIL. These findings suggest that LYPLAL1 genotyping and eating behavior assessments can help predict RYGB surgery success [45].

### 3.8. Phosphatase and Tensin Homolog Gene (PTEN)

A patient with a PTEN mutation achieved a total body weight loss of 39.4% at 1 year, 48.8% at 2 years, and 44.9% at 3 years post-surgery, comparable to a control group of patients with normal genetic test results. The successful weight loss following Sleeve Gastrectomy suggests it could be an effective treatment for obesity in patients with PTEN mutations. Further research on a bigger sample with long-term follow-up is needed [46].

### 3.9. Fatty-Acid-Binding Protein 2 Gene (FABP-2)

Two studies found that the FABP2 rs1799883 genotypes did not affect weight loss or clinical outcomes after MBS. Both wild-type and mutant genotype groups showed similar improvements in BMI, weight, FM, and metabolic parameters, with no significant differences in %EWL at 12 months [42,47].

### 3.10. Cannabinoid Receptor Type 1 Gene (CNR1)

A study of 66 patients found that the G1359A polymorphism in the CB1 receptor did not significantly affect outcomes after biliopancreatic diversion surgery. Both wild-type (G1359G) and mutant (G1359A) groups experienced similar decreases in BMI, weight, waist circumference, blood pressure, glucose, cholesterol, and triacylglycerol levels. The percentage of initial weight loss at one year was also comparable between the groups (33.1% vs. 33.6%) [48].

### 3.11. Leptin Gene (LEP) and Leptin Receptor Gene (LEPR)

One study found that patients with the AA genotype of rs1137101 experienced significantly higher excess weight loss (%EWL) at 12 months (76.5%) and 24 months compared to those with the GG genotype (52.0%). rs8179183 and rs1805094 genotypes showed no significant association [42]. Conversely, another study revealed that patients with the rs1805094 variant of the LEPR gene had a higher initial %EWL at one year (38.9% vs. 29.9%) and greater total weight loss (50.7 kg vs. 37.2 kg) compared to the wild-type group, despite having a higher baseline weight and BMI [49]. Thus, specific genotypes of rs1137101 and rs1805094 are associated with better weight loss outcomes post-surgery.

### 3.12. Glucagon-like Peptide-1 Receptor Gene (GLP-1R)

Patients carrying the rs6923761 GG genotype showed lower BMI, weight, and waist circumference compared to A-allele carriers at 12 and 18 months after MBS. The GG genotype was associated with higher initial weight loss percentages at both 12 months (45.6% vs. 39.8%) and 18 months (49.6% vs. 41.3%) compared to A-allele carriers. Biochemical and cardiovascular improvements were similar across both genotype groups [50].

### 3.13. Apolipoprotein A1 Gene (APOA-I), Apolipoprotein E Gene (APOE), and Adiponectin Gene (ADIPOQ)

In a study involving rs670 polymorphism of the APOA1 gene, both GG and GA/AA groups showed improvements in percent excess weight loss, anthropometric measurements, and biochemical parameters. A-allele carriers (GA/AA) experienced greater reductions in fasting insulin levels at 1, 2, and 3 years post-surgery compared to GG carriers. Similarly, A-allele carriers showed more significant improvements in HOMA-IR levels and increases in HDL-cholesterol levels over the same period [51].

A study that explored associations between APOE polymorphisms and endocrine functions in 62 patients showed that adiponectin levels were significantly lower in the APOE E2 group compared to the E4 group. Adiponectin showed a positive association, while cortisol showed a negative association, across the ordered APOE groups (E2-E3-E4) [52].

In a study exploring the ADIPOQ (rs266729), both genotype groups demonstrated improvements in weight loss and various metabolic markers after MBS. Non-G-allele carriers showed greater reductions in fasting insulin, insulin resistance, total cholesterol, LDL cholesterol, and triglycerides compared to G-allele carriers. Adiponectin levels increased significantly more in non-G-allele carriers over one, two, and three years, particularly in those with the CC genotype [53]. Another study exploring ADIPOQ (rs3774261) revealed decreases in total cholesterol, LDL cholesterol, and triglyceride levels across all genotype groups after MBS. Significant improvements in glucose, insulin, and insulin resistance were observed earlier in subjects with the AA genotype compared to AG and GG genotypes. Adiponectin levels increased significantly in AA-genotype subjects throughout the three-year follow-up, and in AG-genotype subjects at three years. Conversely, GG-genotype subjects did not show significant improvements in these parameters, indicating a delayed response among G-allele carriers of rs3774261 [54]. In a study of 100 patients undergoing RYGB, no association was found with the adiponectin (rs2241766) variant and weight loss after MBS [55].

### 3.14. Interleukin 6 Gene (IL-6)

Two studies on grade II-III obesity patients that underwent LAGB showed that rs1800795 polymorphism of IL-6 provides the opportunity to predict therapeutic response. One study found that rs1800795 is linked to body composition and fluid distribution in obese individuals both before and six months after LAGB surgery. *C*-allele carriers exhibited continued cardiovascular risk and higher FM compared to the reference population. They also showed less weight and FM loss after LAGB and experienced a more pronounced negative impact on bone density, suggesting reduced effectiveness of LAGB in obesity treatment for these genotype carriers [56]. The other study highlighted baseline differences in extracellular water and intra-cellular water between rs1800795 carriers (C+) and non-carriers (C−). LAGB surgery significantly reduced weight and BMI in both groups. Post-surgery, C− carriers showed decreases in weight, BMI, and ECW, with increases in body cell mass (BCM) and phase angle (PA). In contrast, C+ carriers experienced reductions in weight, BMI, ICW, and PA, with increases in ECW and resistance (R). C+ carriers also showed greater reductions in BMI and phase angle compared to C− carriers, suggesting potential utility of IL-6 genotyping for predicting body composition changes following LAGB [57].

### 3.15. Peroxisome Proliferator-Activated Receptor Gamma Coactivator 1-Alpha Gene PGC1a

In a study of 55 patients undergoing Roux-en-Y gastric bypass, those with the rs8192678 polymorphism showed greater improvements in waist/hip ratio, fasting blood glucose, *C*-reactive protein, blood leukocyte count, serum interleukin-6, and carotid artery intima–media thickness compared to Gly/Gly patients. This suggests that the rs8192678 polymorphism may predict better metabolic and inflammatory outcomes, potentially lowering atherosclerotic risk post-surgery [58].

### 3.16. Transmembrane 6 Superfamily 2 Human Gene (TM6SF2), Membrane-Bound O-Acyltransferase Domain-Containing Protein 7 Gene (MBOAT7) and Patatin-like Phospholipase Domain-Containing 3 Gene (PNPLA3)

In a study of 84 obese patients undergoing MBS, those with PNPLA3 rs738409 showed higher hepatic triglyceride content, MRI-detected steatosis, and lower serum glucose levels pre-surgery. After one year, these patients experienced greater weight loss and liver fat reduction compared to those with the wild-type allele. The PNPLA3 mutation and initial steatosis grade were key predictors of MASLD improvement, while TM6SF2 (rs58542926) and MBOAT7 (rs641738) variants were not significant. Thus, PNPLA3 rs738409 may predict better hepatic steatosis outcomes after MBS [59].

### 3.17. Transcription Factor 7-like 2 Gene (TCF7L2)

In a study of 99 morbidly obese patients undergoing RYGB surgery, the TCF7L2 rs7903146 variation influenced the decrease in fasting blood glucose levels over one year. Both groups experienced similar BMI reductions, but T-risk-allele carriers had a smaller decrease in FBG compared to non-carriers. At one year, FBG was higher in T-risk-allele carriers (6.42 vs. 5.36 mmol/L, *p* = 0.022). The TCF7L2 rs7903146 variation affected FBG reduction independently of weight loss [60].

### 3.18. Estrogen Receptor 1 Gene (ESR1)

In a study of 508 patients undergoing MBS (LAGB or LMGB), it was shown that the ESR1 rs712221 polymorphism influenced serum uric acid reduction after MBS. The LMGB group showed a greater reduction in serum uric acid levels compared to the LAGB group (−2.3 vs. −1.2 mg/dL, *p* = 0.002). Additionally, patients with the rs712221 genotype experienced better glycemic control and the greatest reduction in serum uric acid levels post-surgery. This suggests that the type of surgery and the rs712221 polymorphism synergistically affect serum uric acid reduction [61].

### 3.19. Ghrelin Receptor Gene (GHSR), Preproghrelin Gene (GHRL), and CD40 Ligand Gene (CD40L)

A study of 100 obese patients undergoing MBS found that those carrying the T allele of the GHRL rs696217 variant experienced a significantly greater reduction in BMI 52 weeks after RYGB compared to those with the GG genotype. On the other hand, the C allele of the CD40L rs1126535 variant was associated with a lower BMI reduction at week 52. The GHSR rs490683 and GHSR rs27647 variants did not show any difference between carriers and non-carriers of the mutant allele [55]. Another study including GHSR rs572169 and GHRL rs26802 showed no association with weight loss after RYGB [20].

### 3.20. Iodothyronine Deiodinase 2 Gene (DIO2)

A study on 182 obese patients undergoing MBS showed that weight loss and comorbidity improvement were similar across all genetic variations of the DIO2 rs225014 variation. The specific rs225014 variation might be linked to earlier onset of severe obesity, but it does not affect surgical outcomes [62].

### 3.21. Acyl-CoA Synthetase Long Chain Family Member 5 Gene (ACSL5)

In a study of 48 patients undergoing RSG, rs2419621 T-allele carriers experienced greater improvements in EWL%, weight loss, waist circumference, and triglyceride levels 12 months after MBS. Hypertriglyceridemia was eliminated in T-allele carriers by 12 months [63].

### 3.22. GC Vitamin-D-Binding Protein Gene (GC)

In a study of 76 participants, the rs2282679 GC gene variant influenced 25-hydroxyvitamin D levels and insulin resistance after MBS. G-allele carriers had higher basal insulin levels and HOMA-IR and lower 25-hydroxyvitamin D levels compared to non-carriers. These differences persisted throughout the 12-month follow-up. At 12 months, non-G-allele carriers had 25-hydroxyvitamin D levels above 30 ng/dL, while G-allele carriers had levels below 30 ng/dL. This suggests the G allele is associated with lower 25-hydroxyvitamin D levels and greater insulin resistance, impacting the improvement of these parameters after weight loss surgery [64].

### 3.23. Taste 2 Receptor Member 38 Gene (TAS2R38), CD36 Gene (CD36), and Odorant-Binding Protein 2A Gene (OBPIIa)

In a study of 51 obese patients undergoing MBS, taste perception changes were observed. PAV haplotype carriers were more likely to be super-tasters after surgery, while AVI haplotype carriers tended to be non-tasters before surgery. However, these changes were not linked to the TAS2R38 gene. All genotypes showed improved fatty acid taste perception post-surgery, especially those with the non-taster variant (AA). The OBPIIa rs2590498 (A/G) locus was associated with changes in overall taste sensitivity, particularly for sweet and sour tastes. GG carriers had a significantly increased total taste score after surgery, unlike AA or AG carriers [65].

### 3.24. Neuropeptide Y Gene (NPY)

In a study of 147 morbidly obese patients without T2D undergoing MBS, improvements in fasting glucose, insulin, HOMA-IR, and lipid profiles were observed across all genotypes. However, A-allele carriers of the rs16147 NPY gene showed significantly greater reductions in glucose, fasting insulin, and HOMA-IR 1 year after MBS. These results suggest that the A-allele variant is associated with a faster metabolic response after MBS [66].

### 3.25. Brain-Derived Neurotrophic Factor Gene (BDNF)

In a study of 158 severely obese patients undergoing MBS in Spain, researchers focused on the BDNF rs6265 variant and its impact on weight loss over 24 months. They found that patients with the Met allele tended to achieve better weight loss outcomes (%EWL) after surgery, although this was not statistically significant (*p* = 0.056). Patients with the Met allele and no T2D achieved greater %EWL and %EBMIL compared to those with T2D or the Val/Val genotype. These findings suggest that BDNF genotype, combined with T2D status, influences weight loss success following MBS [67].

### 3.26. Clock Circadian Regulator Gene (CLOCK)

In a study involving 375 patients with morbid obesity and 230 controls, the rs1801260 variant showed a protective effect against morbid obesity, while the TT genotype of rs3749474 was strongly associated with morbid obesity. Both SNPs were independently linked to long-term weight loss and weight regain after surgery, regardless of pre-surgery patient characteristics [68].

### 3.27. Calcium/Calmodulin-Dependent Protein Kinase Kinase 2 (CAMKK2)

The study assessed how the CAMKK2 NM_001270486.1, c.1614dup (p.Gly539Argfs*3) mutation affects lipid levels before and 12 months after MBS. Both mutation carriers and non-carriers showed increased HDL cholesterol levels, with non-carriers having a more significant percentage change. Non-carriers also experienced greater reductions in triglyceride levels compared to mutation carriers. These findings highlight that, while both groups benefited from surgery, non-carriers demonstrated more pronounced improvements in lipid metabolism [69].

## 4. Discussion

This systematic review considered fifty-two studies. Our review indicates that there are multiple genetic polymorphisms that have individual effects on weight loss and improvement of obesity-related diseases after MBS. Most of the studies focused on finding associations between the genetic polymorphisms and weight-loss after MBS, but some also explored association with metabolic and cardiovascular comorbidities, as well as eating behavior and taste. The most frequently researched genetic polymorphisms were in the UCP2, MC4R, and FTO genes, each included in eight studies. These genes are prioritized in research due to their well-known roles in energy balance, appetite regulation, and metabolic processes. Based on our narrative synthesis, the UCP2 rs659366 and rs660339 polymorphisms, the FKBP5 rs1360780 polymorphism, and various FTO and MC4R polymorphisms have the biggest potential to be used as predictive markers for the outcome of MBS. Several other polymorphisms, including those in CAT haplotypes, LYPLAL-1, APOA1, APOE, ADIPOQ, PGC-1, MBOAT7, PNPLA3, and TCF7L2, show potential for predicting metabolic improvement outcomes following MBS. However, further research is needed to thoroughly validate and understand their predictive capabilities.

Uncoupling proteins 2 and 3 belong to an anion-carrier protein family located in the inner mitochondrial membrane. UCP2 exhibits widespread expression across various body tissues, including white adipose tissue, whereas UCP3 is predominantly expressed in skeletal muscle [70]. UCP proteins regulate energy expenditure by uncoupling oxidative phosphorylation in mitochondria, dissipating energy as heat. Decreased UCP activity can lead to reduced energy expenditure, contributing to obesity. Additionally, UCPs influence insulin sensitivity and lipid metabolism, linking them to obesity-related conditions like type 2 diabetes and metabolic syndrome [16,71]. Regarding UCP2 rs659366 polymorphism, the studies we found reported opposing conclusions [18,19,20,21]. For the rs660339 polymorphism, it is interesting to note that different bariatric procedures yielded different results, with the T allele showing association with reduced BMI after LAGB, no association after LMGB, and mixed results after RYGB [19,22,23]. Regarding the UCP3 polymorphisms, both studies showed no association with weight loss or clinical outcome after MBS [20,26]. Further studies should be performed on bigger mixed population samples and with a longer follow-up duration in order to provide more accurate and high-quality data.

The predominant cause of non-syndromic genetic obesity often stems from mutations in the MC4R [72]. MC4R is coding the melanocortin-4-receptor which is a G protein-coupled receptor [73] The melanocortin pathway regulates appetite and energy balance through receptors in the brain, influencing food intake and energy expenditure. Dysregulation can lead to obesity by increasing appetite and reducing metabolism. This pathway’s role in adipogenesis and insulin sensitivity also links it to obesity-related diseases like T2D and cardiovascular disorders [16,71]. The studies we found had contradictory results about the association of MC4R polymorphisms and weight loss after MBS. The limitations for some of these studies were the rarity of patients with MC4R mutations, since there is a great heterogeneity of the disorder, and a short period of follow-up [29,30,31].

The FTO gene produces a nuclear protein known as alpha-ketoglutarate-dependent dioxygenase FTO [74]. The FTO gene regulates body weight and adiposity by influencing appetite and energy expenditure through its role in hypothalamic function. Variants associated with FTO increase food intake and decrease satiety, predisposing individuals to obesity. FTO gene variants also correlate with higher risks of T2D and cardiovascular diseases [16,71]. Different FTO polymorphisms were associated with weight loss after MBS, especially after long periods of follow-up [37,38,39]. Other studies showed no association, but they were performed on a smaller sample and with shorter follow-up [40,42]. In patients undergoing RSG, those without the A allele experienced better anthropometric and insulin level improvements, reducing diabetes risk after 12 months [43]. Patients with the three polymorphisms rs9939609, rs9930506, and rs1421085 had higher insulin and HOMA-IR [28]. It is important to assess pre-surgery vitamin D levels in future studies, since it has been shown they affect weight loss in certain carriers [41].

Studies investigating FKBP5 determined that the T allele was associated with weight loss [35,36] with worse results in older males who have undergone LSG [36]. Future longitudinal studies with larger sample sizes and longer follow-up period might shed more light on the role of FKBP5 variability and its interaction with variables, such as stress, surgery, and sex.

Some genetic polymorphisms were connected to eating habits, such as 5-HT2C [20] and LYPAL-1 [45], and taste changes after MBS, such as TAS2R38, CD36, and OBPII2 [65]. Ghrelin, produced in the stomach, increases appetite by signaling hunger to the brain. Leptin, released by fat cells, suppresses appetite and regulates energy balance. Imbalances, such as elevated ghrelin or leptin resistance, can contribute to overeating and obesity, highlighting their crucial roles in appetite regulation and metabolic health [75]. It was shown that specific genotypes of rs1137101 and rs1805094 of the leptin gene and rs696217 of GHRL are associated with better weight loss outcomes after MBS [42,49,55]. Others, such as CAT haplotypes [44], GLP-1R [50], APOA-1 [51], ADIPOQ [53,54,55], PGC1a [58], TCF7 L2 [60], ESR1 [61], IL-6 gene [56,57], ACSL5 [63], CG [64], NPY [66], and CAMKK2 [69], influenced metabolic, inflammatory, and cardiovascular parameters after MBS. The GLP-1R gene encodes the GLP-1 receptor, crucial in glucose metabolism and satiety regulation [71]. One study showed that the rs6923761 variant was associated with weight loss after BMS [50]. GLP-1 receptor agonists enhance insulin secretion, delay gastric emptying, and reduce appetite, aiding weight loss in obesity and improving metabolic parameters in related diseases like type 2 diabetes [76]. Larger in-depth longitudinal studies with representative samples and a longer follow-up should be performed to further investigate the role of the polymorphisms and these genes on the outcome of different types of MBS.

Only one study was found that investigated the outcome of MBS in obese patients diagnosed with MASLD. It showed that patients with PNPLA3-associated steatohepatitis show better improvement of hepatic steatosis after MBS as compared to carriers of PNPLA3 wild-type alleles; consequently, PNPLA3 rs738409 is a good prognostic factor for MASLD patients undergoing MBS. On the other hand, TM6SF2 rs58542926 or MBOAT7 rs641738 did not have major effect on hepatic steatosis, even if carriers of the MBOAT7 minor allele presented with increased serum glucose, triglycerides, and cholesterol before surgery [59]. However, it should be taken into consideration that the sample size was small and the follow-up was only 12 months.

While this review provides valuable insights, it is important to acknowledge several limitations. The inclusion criteria restrict the review to English-language studies that are freely available, potentially excluding valuable research in other languages or behind paywalls. While the quality and risk of bias were assessed, the reliance on narrative synthesis without statistical pooling may introduce subjectivity, as it lacks the statistical rigor of a meta-analysis. The included studies varied in sample size, genetic variants studied, methodologies, and types of bariatric surgery procedures, which may introduce heterogeneity and affect the generalizability of the results. Additionally, many studies had short follow-up periods, limiting our understanding of the long-term impacts of genetic factors on bariatric surgery outcomes.

Future research should focus on developing polygenic scores in a large, diverse population, because the aggregated effect of the multiple genetic variants, as well as the heterogeneity of the population, will provide a better prognostic tool for the outcomes of MBS. Polygenic scores should extend beyond predicting weight loss to also include effects on metabolism, cardiovascular conditions, and eating behaviors. Key analyses should involve large-scale genome and exome studies to identify relevant polymorphisms. Prioritizing diverse cohorts with extended follow-up will validate findings and explore additional variants. Expanding genetic analysis and study diversity will help create accurate prognostic tools and tailor interventions to individual genetic profiles.

## 5. Conclusions

This systematic review examined 52 studies focusing on genetic polymorphisms in UCP2, MC4R, and FTO genes, which were the most frequently researched. Findings indicated varied associations between these genes and weight loss outcomes following MBS. The UCP2 rs659366 and rs660339 variants showed inconsistent results across different MBS types. MC4R polymorphisms also yielded mixed results, limited by small sample sizes and short follow-up periods. The FTO gene’s impact varied with surgery type and duration, with some polymorphisms linked to weight loss differences, insulin levels, and diabetes risk. Additional genetic polymorphisms were connected to eating habits, metabolic changes, and cardiovascular parameters. Despite these insights, study limitations like sample size, methodology variability, and short follow-up periods highlight the need for larger, diverse cohorts with extended follow-ups to better understand genetic influences on MBS outcomes.

## Figures and Tables

**Table 1:** Summary of studies included in the systematic review.

Genes	Polymorphism	Population Sample (n)	Procedure	Follow-Up	Main Results	Reference
UCP2	rs659366	Italian patients with grade II or III obesity (164)	LAGB	6 months	A/A genotype was associated with increased weight loss 6 months after LAGB (adjusting for age, gender, and baseline BMI).	[18]
		Brazilian patients with grade II or III obesity (150)	RYGB	12 months	A allele was associated with increased weight loss and FFM 12 months after RYGB (adjusting for age, gender, and initial weight).	[19]
		Brazilian patients with grade II or III obesity (351)	RYGB	12 months	No association with % of excess of weight loss 12 months after RYGB.	[20]
		Patients with grade II or III obesity (186)	RYGB	6, 12, and 18 months	The UCP2 2866A/A genotype is associated with higher BMI, more excess weight, and lower EWL% 18 months after MBS.	[21]
	Ins/Del	Patients with grade II or III obesity (186)	RYGB	6, 12, and 18 months	Patients with the Ins allele had a smaller decrease in BMI 12 months after MBS compared to those with the Del/Del genotype.	[21]
	rs660339	Taiwanese patients with grade II or III obesity (520)	LAGB (149) LMGB (371)	6 months	T allele was associated with reduced BMI 6 months after LAGB. No association with BMI changes after LMGB.	[22]
		Taiwanese patients with grade III obesity (304)	LAGB (77) LMGB (227).	12 and 24 months	T allele was associated with reduced BMI 12 and 24 months after LAGB. No association with BMI changes after LMGB.	[23]
		Brazilian patients with grade II or III obesity (150)	RYGB	12 months	T allele was associated with higher weight loss and FFM 12 months after RYGB (adjusting for age, gender, and initial weight).	[19]
		Brazilian patients with grade II and III obesity (143)	RYGB	3 years	No association with weight loss 3 years after RYGB.	[24]
		Brazilian patients with grade II or III obesity (351)	RYGB	12 months	No association with % of excess of weight loss 12 months after RYGB.	[20]
	rs659366 rs660339	Brazilian patients with grade II or III obesity (150)	RYGB	12 months	Patients with at least 1 rare allele for polymorphisms and with at least 1 rare allele for both polymorphisms together (haplotype) present greater energy and carbohydrate intake even after adjustment for sex, age, and weight.	[25]
UCP3	rs1800849	Brazilian patients with grade II or III obesity (351)	RYGB	12 months	No association with % of excess of weight loss 12 months after RYGB.	[20]
		Taiwanese patients with grade III obesity (40)	BPD	12 months	No association with weight loss or clinical outcomes 12 months after BPD.	[26]
5-HT2C	rs3813929	Brazilian patients with grade II or III obesity (351)	RYGB	12 months	The TT genotype predicts greater percentage of %EWL among female patients.	[20]
MC4R	rs17782313	Patients with grade II or III obesity (217)	RYGB	60 months	Women carrying this polymorphism present higher presurgical BMI and tend to maintain BMI > 35 kg/m^2^, which characterizes treatment failure.	[27]
		Patients with grade II or III obesity (101)	RYGB	4 to 8 years	No association was found betwen the MC4R polymorphism and TBWL, post-surgery weight, and BMI after MBS.	[28]
	NM_005912.2 c. 812G>T (p. Cys271Phe) NM_005912.2 c. 919C>T (p. Gln307stop) NM_005912.2 c. 706C>T (p. Arg236Cys)	Patients with grade III obesity (92)	RYGB	1, 3, 6, 9, and 12 months	Patients with heterozygous MC4R mutations had a similar rate of weight loss and %EWL after RYGB as patients without MC4R mutations.	[29]
	functional mutations rs2229616 rs52820871 rs17782313 rs34114122	Patients with grade II or III obesity (648)	LAGB and RYGB	3, 6, and 12 months	Heterozygous mutations near and in the MC4R gene, either leading to a reduced receptor function or not, did not affect weight loss and body FM after MBS.	[30]
	rs52820871 NM_005912.2 c.594C>T (p. Ile198Ile)	Patients with grade II or III obesity (370)	LAGB	65 months	No association between MC4R defects and higher complication rate following LAGB.	[31]
	rs52820871 rs2229616	Patients with grade III obesity (1433)	RYGB	Up to 48 months	Individuals carrying the rs52820871 common allele are predisposed to better clinical outcome, reduced risk of T2D, and better weight loss during diet and surgical interventions.	[32]
	rs199862517 rs13447329 rs13447332 rs2229616 rs52820871 NM_005912.3 c. 544T>C (p. Phe51Leu) NM_005912.3 c.991A>G (p. Met200Val) NM_005912.3 c.1419 A>G (p. 3′UTRl)	Patients with grade II or III obesity (300)	LAGB	36 ± 3 months	Carriers of MC4R gene variants exhibit an aggressive form of BED associated with the metabolic syndrome and gastrointestinal pathology, with poorer response to massive sustained weight loss from food restriction after LAGB.	[33]
	rs79783591	Mexican patients with grade II or III obesity (206)	RYGB	6 months	%EWL was similar in non-carriers and heterozygous patients 6 months after MBS, while it was lower in the Asn269Asn homozygous patient.	[34]
FKBP5	rs1360780	Patients with grade II or III obesity (42)	RYGB	Up to 26 months	The T allele is associated with weight loss.	[35]
		Patients with grade II or III obesity (151)	RYGB (94) LSG (57)	24 months	FKBP5 rs1360780 genotype has specific effects on weight loss outcomes after MBS depending on sex, age, and type of surgery, suggesting worse results in older males carrying the T allele who have undergone LSG.	[36]
FTO	rs16945088	Patients with grade II or III obesity (1443)	VBG (1368) LAGB (377) RYGB (265)	6 years	Associated with maximum weight loss after LAGB.	[37]
	rs9939609	Patients with grade II or III obesity (119)	BPD	3, 9, and 12 months	Higher initial weight loss at 3 months in TT variant of FTO gene. Weight loss at 9 and 12 months of BPD was similar in both genotypes with a significant improvement in biochemical parameters and cardiovascular co-morbidities.	[38]
		Patients with grade II or III obesity (146)	RYGB	5 years	FTO-SNP (AA or AT genotypes) does not influence weight until 2 years after surgery. Weight loss was lower in FTO-SNP group starting 2 years after surgery. Weight regain was higher and sooner in FTO-SNP group.	[39]
		Patients with grade III obesity (74)	LSG	6 months	There was no association between FTO gene polymorphism and weight loss 6 months after LSG.	[40]
		Patients with grade III obesity (210)	RYGB	2 years	Pre-surgery vitamin D levels are crucial for subsequent weight loss, especially in those who carry two copies of the A-allele.	[41]
		Brazilian patients with grade II or III obesity (105)	RYGB	3, 6, 12, 24 months	Did not show any effect on weight loss or clinical outcomes after MBS.	[42]
		Patients with grade II or III obesity (95)	RSG	3, 6, and 12 months	Patients without the A allele of the FTO rs9939609 variant had better improvement of anthropometric and insulin levels after MBS, which decreased the risk for diabetes after 12 months.	[43]
		Patients with grade II or III obesity (101)	RYGB	4 to 8 years	rs9939609 and rs1421085 were associated with higher weight after MBS. rs9930506 and rs1421085 were associated lower TBWL after MBS. Patients with these three polymorphisms also had higher insulin and HOMA-IR.	[28]
	rs9930506					
	rs1421085,					
CAT haplotype	CAT1 CAT2	Patients with grade II or III obesity (294)	RYGB (256) LAGB or LSG (38)	3, 6, and 12 months	CAT1 and CAT2 catalase gene promoter haplotypes are implicated in metabolic differences observed in overfeeding or fasting context. CAT1 displays a more beneficial impact on metabolic and cardiovascular parameters (obesity grade, blood pressure level, inflammation level) after MBS.	[44]
LYPLAL-1	rs4846567	Patients with grade I, II, or III obesity (251)	RYGB	2 years	The gene variant rs4846567 is associated with the strength of eating behavior before surgery and the magnitude of excess BMI loss after RYGB surgery.	[45]
PTEN	rs398123317	Female patient with morbid obesity (1)	LSG	1, 2, and 3 years	Positive weight loss results after LSG.	[46]
FABP-2	rs1799883	Patients with grade III obesity (41)	BPD	1 year	Polymorphism Ala54Thr of FABP did not have an effect on weight loss or clinical outcomes after MBS.	[47]
		Brazilian patients with grade II or III obesity (105)	RYGB	3, 6, 12, 24 months	Did not show any effect on weight loss or clinical outcomes after MBS.	[42]
CNR1	rs1049353	Patients with grade III obesity (66)	BPD	3, 9, 12 months	Polymorphism G1359A in the CB1 receptor did not have a significant effect on biochemical and anthropometric improvements after MBS.	[48]
LEP LEPR	rs1805094	Patients with grade III obesity (41)	BPD	3, 9, 12 months	Weight loss was higher in mutant group (Lys656Asn and Asn656Asn) than wild-type group (Lys656Lys) after MBS. Carriers of the allelic variant (Asn) had higher basal weight.	[49]
	rs8179183 rs1805094	Brazilian patients with grade II or III obesity (105)	RYGB	3, 6, 12, 24 months	Did not show any effect on weight loss or clinical outcomes after MBS.	[42]
	rs1137101	Brazilian patients with grade II or III obesity (105)	RYGB	3, 6, 12, 24 months	There is a different evolution of weight loss in carriers of the rs1137101 after MBS. The AA genotype seems to be associated with a higher weight loss.	[42]
GLP-1R	rs6923761	Patients with grade III obesity (137)	BPD	3, 9, 12, and 18 months	Higher weight loss after MBS in GG variant than A-allele carriers. The biochemical parameters and cardiovascular comorbidity rates improved similarly in both genotypes.	[50]
APOA-1	rs670	Patients with grade III obesity (63)	BPD	1, 2, 3 years	The variant rs670 of the APOA1 gene showed important effects on HDL-cholesterol, HOMA-IR, and insulin resistance after derivation biliopancreatic during 3 years. A-allele carriers showed high levels of HDL cholesterol.	[51]
APOE	E2 (ε2ε2 + ε2ε3) E3 (ε3ε3 + ε2ε4) E4 (ε3ε4 + ε4ε4)	Patients with grade II or III obesity (62)	RYGB (52) SG (10)	12 months	APOE polymorphism is associated with endocrine effects on body weight and metabolic functions in morbidly obese patients. The ε4 allele was positively associated with adiponectin and negatively associated with cortisol.	[52]
ADIPOQ	rs266729	Patients with grade III obesity (149)	BPD	3 years	Non-G allele of ADIPOQ gene variant (rs266729) is associated with increases in adiponectin levels and better improvement of LDL cholesterol, triglycerides, insulin, and homeostasis model assessment of insulin resistance after BPD massive weight loss than G-allele carriers.	[53]
	rs3774261	Patients with grade III obesity (149)	BPD	1, 2, and 3 years	Patients with the G allele at rs3774261 exhibited slower improvements in glucose metabolism, adiponectin levels, and the adiponectin/leptin ratio after MBS.	[54]
	rs2241766	Patients with grade II or III obesity (100)	RYGB	6 and 52 weeks	No association was found with the adiponectin (rs2241766) variant and weight loss after MBS.	[55]
IL-6	rs1800795	Italian patients with grade II or III obesity (40)	LAGB	6 months	Association of IL-6 variant with fluid distribution, at baseline, and FM and bone density loss in obese subjects at 6 months follow-up after LAGB surgery. LAGB was less effective if the subjects were carrying risk genotypes, C(−) carriers, for obesity.	[56]
		Italian patients with grade II or III obesity (20)	LAGB	3 months	Significant higher reductions in BMI and Xc/H were observed in C(+) with respect to C(−) carriers. The 174 G > C polymorphism of IL-6 provides the opportunity to predict therapeutic response of obese subjects, in terms of body composition outcomes, through bioelectrical evaluation.	[57]
PGC1a	rs8192678	Obese patients eligible for surgery (55)	RYGB	1 year	Gly482Ser polymorphism may predict a more favorable metabolic and inflammatory outcome for obese patients submitted to MBS.	[58]
TM6SF2	rs58542926	Patients with grade II or III obesity (84)	RYGB (43) LSG (41)	12 months	The improvement of steatosis after surgery was not affected by the presence of TM6SF2 variants.	[59]
MBOAT7	rs641738	Patients with grade II or III obesity (84)	RYGB (43) LSG (41)	12 months	The MBOAT7 polymorphism was associated with increased triglycerides, total cholesterol, LDL, and glucose levels in serum.	[59]
PNPLA3	rs738409	Patients with grade II or III obesity (84)	RYGB (43) LSG (41)	12 months	PNPLA3 p.I148M variant is a good prognostic factor for MASLD patients undergoing MBS.	[59]
TCF7L2	rs7903146	Patients with grade III obesity (99)	RYGB	1, 3, 6, and 12 months	TCF7 L2 gene variation affected the decrease in fasting blood glucose after RYGB in obese patients with T2D, independently of weight loss.	[60]
ESR1	rs712221	Han Chinese patients with grade II or III obesity (508)	LAGB (164) LMGB (344)	12 months	ESR1 polymorphism synergistically with surgery type, insulin sensitivity, and protein intake is associated with reduction in serum uric acid levels after MBS.	[61]
GHSR	rs490683	Patients with grade II or III obesity (100)	RYGB	6 and 52 weeks	The rs490683 SNP for ghrelin did not show any difference between carriers and non-carriers of the mutant allele regarding weight loss after MBS.	[55]
	rs572169	Brazilian patients with grade II or III obesity (351)	RYGB	12 months	No association with % of excess of weight loss 12 months after RYGB.	[20]
GHRL	rs696217 rs27647	Patients with grade II or III obesity (100)	RYGB	6 and 52 weeks	Carrying a G-to-T substitution in rs696217 (preproghrelin gene) seems to mark a successful weight loss outcome.	[55]
	rs26802	Brazilian patients with grade II or III obesity (351)	RYGB	12 months	No association with % of excess of weight loss 12 months after RYGB.	[20]
CD40L	rs1126535	Patients with grade II or III obesity (100)	RYGB	6 and 52 weeks	Rs1126535 C allele (CD40L gene) may predict a worse response to MBS.	[55]
DIO2	rs225014	Patients with grade II or III obesity (182)	RYGB (35) OAGB (51) SG (74) AGB (22)	6 and 12 months	The DIO2p.Thr92Ala polymorphism does not affect obesity severity or complications but may lead to earlier onset of complicated obesity. It also does not impact weight loss or remission of comorbidities after MBS.	[62]
ACSL5	rs2419621	Patients with grade III obesity (48)	RSG	3, 6, and 12 months	The ACSL5 gene variant (rs2419621) is associated with better improvement in adiposity and triglyceride levels in subjects with the T allele after MBS.	[63]
GC	rs2282679	Patients with grade II or III obesity (76)	RSG	3, 6, and 12 months	The GC gene variant (rs2282679) is associated with low vitamin D levels and insulin resistance. The G allele also reduced improvement in vitamin D levels and insulin resistance after 12 months of weight loss.	[64]
TAS2R38	rs713598 rs1726866 rs10246939	Caucasian patients with grade II or III obesity (51)	RYGB (30) SG (21)	1 and 6 months	Patients with a PAV haplotype were more likely to be classified as a super-taster after MBS than before while patients with the AVI haplotype were more likely to be classified as a non-taster before MBS than after. Changes in bitter taste perception after MBS are not caused by the TAS2R38 gene.	[65]
CD36	r1761667				The improvement on fatty acid taste after MBS was not related with the CD36 locus. All genotypes tasted fatty acids better after MBS, especially those homozygous for the non-taster variant (AA).	
OBPIIa	rs2590498				The OBPIIa (A/G) locus was associated with the variations in the overall taste sensitivity or sensitivity to sweet and sour tastes after MBS. GG carriers had a significantly increased total taste score after MBS, while AA or AG carriers did not.	
NPY	rs16147	Patients with grade III obesity (147)	BPD	1, 2, 3, and 4 years	The A allele of this genetic variant might be associated with early modulations in glucose, insulin, and HOMA-IR levels following substantial weight loss after MBS.	[66]
BDNF	rs6265	Patients with grade II or III obesity (158)	RYGB (99) SG (59)	1, 3, 6, 12, and 24 months	Met-allele carriers have greater weight loss after MBS than Val/Val carriers. Pre-existing T2D appears to attenuate the beneficial effect.	[67]
CLOCK	rs3749474 rs1801260 rs4580704	Patients with grade II or III obesity (375)	SG (60) RYGB (205) SADI-S (85) BPD-DS (25)	5 to 8 years	rs1801260 A-allele carriers and rs3749474 T-allele carriers were associated with a worse weight response and a higher risk of morbid obesity after MBS.	[68]
CAMKK2	NM_001270486.1, c.1614dup (p.Gly539Argfs*3)	Patients with grade II or III obesity (71)	Not specified	1, 3, 6, and 12 months	MBS improved metabolism in all patients, but non-carriers of the CAMKK2 mutation had higher HDL and lower triglycerides compared to carriers.	[69]

Abbreviations in table: %EWL—percentage of excess weight loss; 5-HT2C—5-hydroxytryptamine receptor 2C gene; ACSL5—Acyl-CoA Synthetase Long Chain Family Member 5 gene; ADIPOQ—adiponectin gene; APOA-1—apolipoprotein A1 gene; APOE—apolipoprotein E gene; BDNF—Brain Derived Neurotrophic Factor gene; BMI—body mass index; BPD—biliopancreatic diversion; CAMKK2—Calcium/Calmodulin-Dependent Protein Kinase Kinase 2; CAT haplotype—catalase haplotype; CB1 receptor—cannabinoid receptor type 1; CD36—CD36 gene; CD40L—CD40 ligand gene; CLOCK—clock circadian regulator gene; CNR-1—cannabinoid receptor type 1 gene; DIO2—Iodothyronine deiodinase 2 gene; T2D—diabetes mellitus type 2; ESR-1—estrogen receptor 1 gene; FABP-2—fatty-acid-binding protein 2; FFM—fat-free mass; FKBP5—FK506-binding protein 5 gene; FM—fat mass; FTO—fat mass and obesity-associated protein gene; GC—GC Vitamin-D-Binding Protein gene; GHRL—preproghrelin gene; GHSR—ghrelin receptor gene; GLP-1R—glucagon-like peptide-1 receptor gene; HDL—high-density lipoprotein; HOMA-IR—Homeostatic Model Assessment for Insulin Resistance; IL-6—interleukin 6; LAGB—laparoscopic adjustable gastric banding; LDL—low-density lipoprotein; LEP—leptin gene; LEPR—leptin receptor gene; LMGB—laparoscopic mini gastric bypass; LSG—laparoscopic sleeve gastrectomy; LYPLAL-1—lysophospholipase-like 1 gene; MBOAT7—membrane-bound O-acyltransferase domain-containing protein 7 gene; MBS—metabolic and bariatric surgery; MC4R—melanocortin 4 receptor gene; NPY—Neuropeptide Y gene; OBPIIa—Odorant-Binding Protein 2A gene; PGC1a—peroxisome proliferator-activated receptor gamma coactivator 1-alpha gene; PNPLA3—patatin-like phospholipase domain-containing 3 gene; PTEN—phosphatase and tensin homolog gene; RSG—sobotic sleeve gastrectomy; RYGB—Roux-en-Y gastric bypass; SG—sleeve gastrectomy; TAS2R38—taste 2 receptor member 38 gene; TCF7 L2—transcription factor 7-like 2 gene; TM6SF2—transmembrane 6 superfamily 2 human gene; UCP2—uncoupling protein 2 gene; UCP3—uncoupling protein 3 gene; VBG—vertical banded gastroplasty.

## Data Availability

Data sharing not applicable.

## Abbreviations

%EWL	percentage of excess weight loss
5-HT2C	5-hydroxytryptamine receptor 2C gene
ADIPOQ	adiponectin gene
ACSL5	Acyl-CoA Synthetase Long Chain Family Member 5 gene
AGB	adjustable gastric banding
APOA-1	apolipoprotein A1 gene
APOE	apolipoprotein E gene
BDNF	Brain Derived Neurotrophic Factor gene
BMI	body mass index
BPD	biliopancreatic diversion
DALY	disability-adjusted life year
MBS	metabolic and bariatric surgery
CAMKK2	Calcium/Calmodulin-Dependent Protein Kinase Kinase 2
CAT haplotype	catalase haplotype
CB1 receptor	cannabinoid receptor type 1
CD36	CD36 gene
CD40L	CD40 ligand gene
CLOCK	clock circadian regulator gene
CNR1	cannabinoid receptor type 1 gene
DIO2	Iodothyronine deiodinase 2 gene
T2D	diabetes mellitus type 2
ESR1	estrogen receptor 1 gene
FABP-2	fatty-acid-binding protein 2 gene
FKBP5	FK506-binding protein 5 gene
FM	fat mass
FTO	fat mass and obesity-associated protein gene
GC	GC Vitamin-D-Binding Protein gene
GHRL	preproghrelin gene
GHSR	ghrelin receptor gene
GLP-1R	glucagon-like peptide-1 receptor gene
GWAS	genome wide association studies
HDL	high density lipoprotein
HOMA-IR	Homeostatic Model Assessment for Insulin Resistance
IL-6	interleukin 6
LAGB	laparoscopic adjustable gastric banding
LEP	leptin gene
LEPR	leptin receptor gene
LGALS3	galectin-3 gene
LMGB	laparoscopic mini gastric bypass
LSG	laparoscopic sleeve gastrectomy
LYPLAL-1	lysophospholipase-like 1 gene
MBOAT7	membrane-bound O-acyltransferase domain-containing protein 7 gene
MC4R	melanocortin 4 receptor gene
MASLD	metabolic dysfunction-associated steatotic liver disease
NPY	Neuropeptide Y gene
OBPIIa	Odorant-Binding Protein 2A gene
PGC1a	peroxisome proliferator-activated receptor gamma coactivator 1-alpha gene
PNPLA3	patatin-like phospholipase domain-containing 3 gene
PTEN	phosphatase and tensin homolog gene
RSG	robotic sleeve gastrectomy
RYGB	Roux en Y gastric bypass
SG	sleeve gastrectomy
TAS2R38	taste 2 receptor member 38 gene
TCF7L2	transcription factor 7-like 2 gene
TM6SF2	transmembrane 6 superfamily 2 human gene
UCP2	uncoupling Protein 2 gene
UCP3	uncoupling Protein 3 gene
VBG	vertical banded gastroplasty
VLDL	very low-density lipoprotein
